# Systematic Understanding of the Mechanism of Salvianolic Acid A via Computational Target Fishing

**DOI:** 10.3390/molecules22040644

**Published:** 2017-04-17

**Authors:** Shao-Jun Chen, Ming-Chao Cui

**Affiliations:** Department of Traditional Chinese Medicine, Zhejiang Pharmaceutical College, 888 Yinxian Avenue Eastern Section, Ningbo 315100, China; cuimc@mail.zjpc.net.cn

**Keywords:** Danshen, druggability, network pharmacology, salvianolic acid A, target fishing

## Abstract

Salvianolic acid A (SAA) is one of the most abundant water-soluble and potent anti-oxidative compounds isolated from Danshen, a traditional Chinese medicine. A systematic overview of its mechanism of action is yet to be performed. In the present study, the druggability of SAA was measured using the TCMSP server, and potential targets of SAA were identified by PharmMapper and DRAR-CPI. Intersecting targets were then assessed by GeneMANIA and GO pathway analysis, and drug-target-pathway networks were constructed to give a visual view. The results showed that SAA has good druggability, and 13 putative protein targets were identified. Network analysis showed that these targets were associated with cancer, metabolism and other physiological processes. In summary, SAA is predicted to target multiple proteins and pathways to form a network that exerts systematic pharmacological effects.

## 1. Introduction

**Figure 1:**
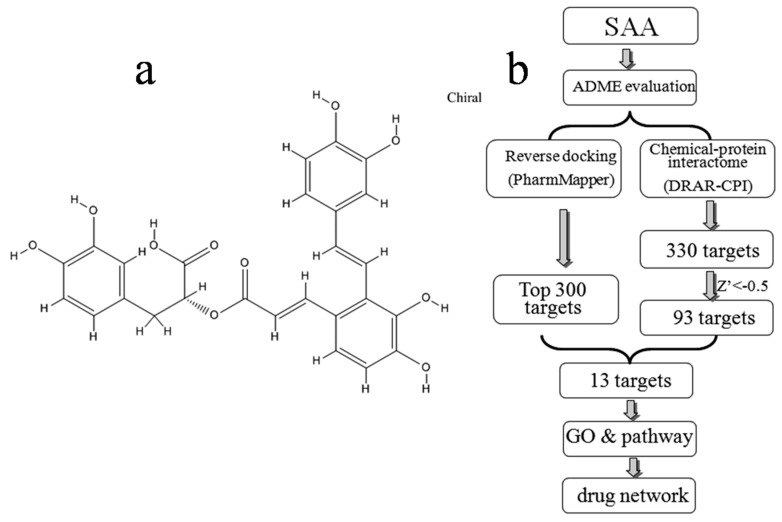
(**a**) Chemical structure of SAA downloaded from the PubChem database (CID: 5281793); (**b**) Pipeline for the identification of putative SAA targets that integrates ADME evaluation, reverse docking, chemical-protein interactome, GO, and pathway analyses, and network construction.

Traditional Chinese medicine (TCM) and natural products in general are the best source of active compounds for drug discovery [1,2]. Salvianolic acid A (SAA; Figure 1a) is one such example, and is one of the most abundant water-soluble components extracted from Danshen (red sage, or *Radix Salvia miltiorrhiza*), a TCM that has been widely used to treat cardiovascular diseases for hundreds of years [3,4]. This compound is the most potent anti-oxidative agent among those isolated from Danshen [5,6]. Furthermore, SAA is of pharmacological relevance due to its antiplatelet and antithrombosis activities, and it also improves microcirculation. Furthermore, anti-inflammation and antioxidant, myocardial ischemic protection, antithrombotic, neuroprotection and anti-fibrosis activities have been reported, and it can prevent diabetes and associated complications [4,6]. For such naturally occurring compounds, knowledge of the toxic or medicinal properties often long predates precise knowledge of targets or mechanisms [1], and there remains much to learn about SAA.

Adoption of computation methodologies for the repositioning of drug molecules in receptors is becoming mainstream because it can save time, money and labour [7,8]. Computational target fishing in particular can improve drug discovery and drug design processes and thereby address unmet medical needs [9]. In our previous work, we identified the potential target of capsaicin and tanshinone IIA using computational tools [10,11]. As an active compound from TCM, SAA is similarly well suited to this type of analysis.

In the present study, the druggability of SAA was first evaluated using the TCMSP server. Potential targets were predicted by both computational reverse docking and chemical-protein interactome analysis, and overlapping targets identified using both approaches were chosen for further investigation based on gene ontology (GO) and pathway analysis. Finally, we constructed a drug-target network to provide a systematic overview of potential targets and mechanisms of action for SAA. An overview of the experimental procedures for SAA target prediction is shown in Figure 1b.

## 2. Results

### 2.1. ADME-Related Properties of SAA

TCMSP provides information on important ADME-related properties such as human oral bioavailability (OB), drug-likeness (DL), Caco-2 permeability (Caco-2), blood-brain barrier (BBB) permeability and Lipinski’s rule of five (MW, AlogP, TPSA, Hdon, Hacc) [12]. ADME-related properties of ASS were investigated in depth by TCMSP (Table 1). Notably, the DL of SAA was calculated to be 0.7 (Table 1).

### 2.2. Identification of Potential Targets

Potential targets were predicted using two different approaches as described in the methods. Figure 1b shows the top 300 potential protein targets of SAA from all 7302 pharmacophore models obtained using PharmMapper, as well as the potential 330 targets identified using DRAR-CPI. In a DRAR-CPI job, a Z′-score threshold of −0.5 indicates favourable targets, and 93 targets met this requirement. Finally, to improve the specificity, a total of 13 potentially interacting proteins identified in both sets of results were selected for further investigation (Table 2).

### 2.3. Analysis by GeneMANIA

**Figure 2:**
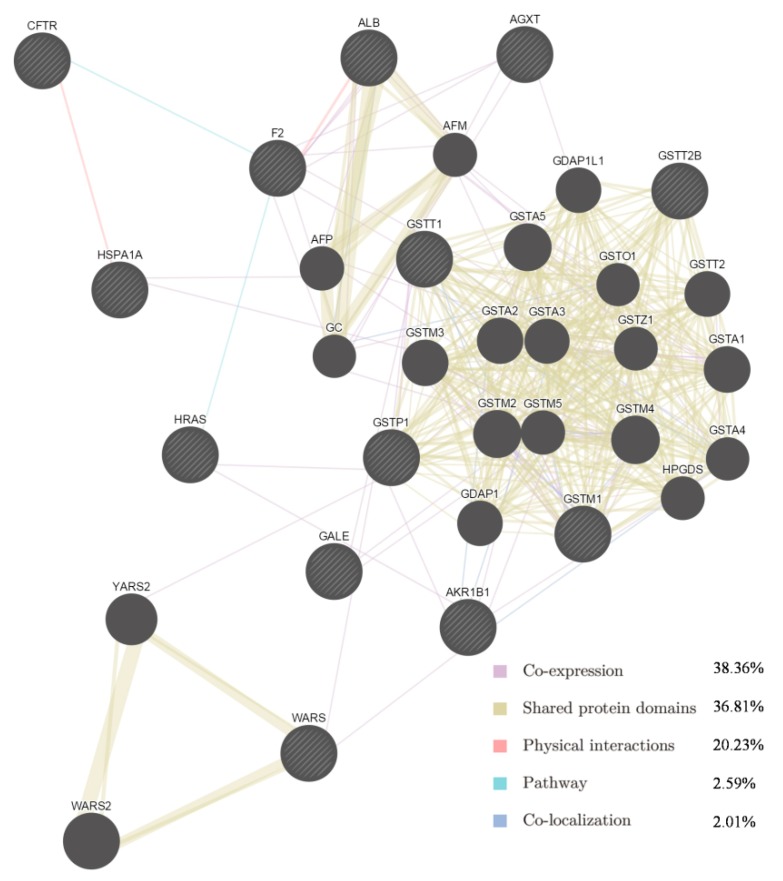
Network of potential SAA targets. Black protein nodes indicate target proteins, and different connecting colours represent different correlations. Functional association of targets was analysed using GeneMANIA. Genes in black circles were submitted as query terms in searches. Grey circles indicate genes associated with query genes.

Among the 13 targets and their interacting proteins, it was found that 38.36% displayed similar co-expression characteristics, and 36.81% shared the same protein domain. Other results including physical interactions, pathways and co-localisation are also shown in Figure 2.

### 2.4. GO and Pathway Analysis, and Network Construction

**Figure 3:**
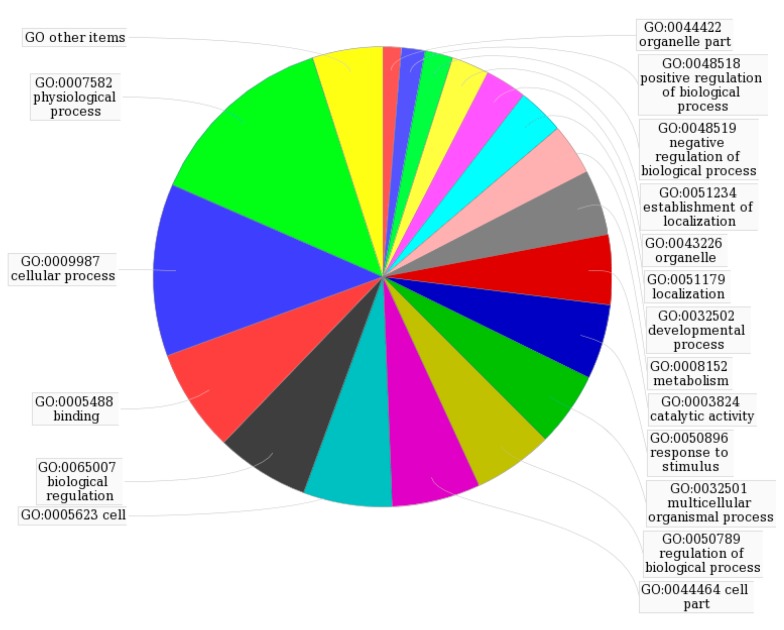
GO map of putative targets following second-level analysis by MAS 3.0.

To further investigate the 13 identified targets, analysis of interaction network regulation was performed using MAS 3.0. As shown in Figure 3 and Table 3, the top five functions were physiological process (GO:0007582), cellular process (GO:0009987), binding (GO:0005488), biological regulation (GO:0065007) and cell (GO:0005623), which together accounted for 45%. As shown in Supporting Table S1, the 13 targets participate in 44 KEGG pathways including glutathione metabolism, metabolism of xenobiotics by cytochrome P450, galactose metabolism, prostate cancer, regulation of actin cytoskeleton and MAPK signalling.

**Figure 4:**
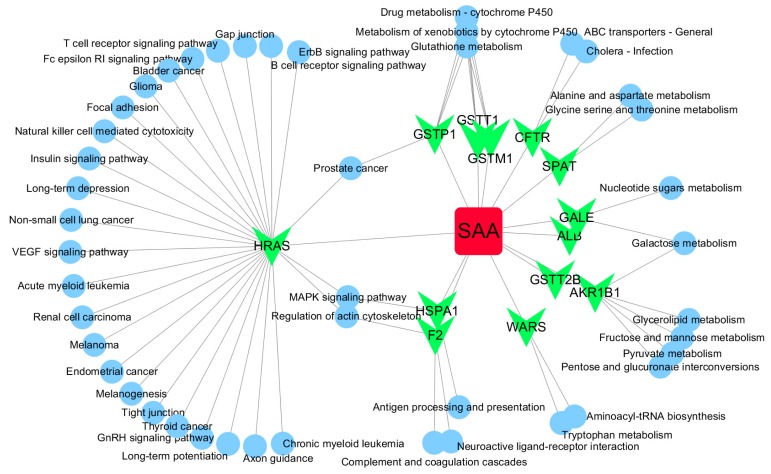
SAA-target-pathway network. Red diamond = SAA, magenta triangle = target proteins, green circles = pathway.

Based on target fishing and pathway analysis, an entire network was constructed using Cytoscape 3.0. As shown in Figure 4, the interaction network has 59 nodes and 68 edges. The red oblong, green inverted triangles and blue circles correspond to SAA, target proteins and pathways, respectively.

## 3. Discussion

Poor pharmacokinetics and toxicity are significant causes of costly late-stage failures in drug development, and it is increasingly accepted that these areas should be prioritised in the drug discovery process [13]. In silico approaches can improve our ability to predict and model pharmacokinetic, metabolic and toxicity endpoints, thereby streamlining and accelerating the drug discovery process [13].

Lipinski’s “rule of 5” can identify several critical properties that should be considered for compounds with oral delivery in mind [13,14]. They are the molecular weight (MW) < 500 daltons (Da), the calculated LogP (CLogP) < 5 (or MlogP > 4.15), number of hydrogen-bond donors < 5 and number of hydrogen-bond acceptors < 10 [13,14]. Now, the rule of five is commonly referred to as a “druglike” measure and guideline in drug lead optimisation [13,15]. As listed in Table 1, SAA's properties meet the requirements, which means SAA is a good candidate for drug discovery.

The concept of DL, established from analysis of physiochemical properties or/and structural features of existing small molecule drugs and/or drug candidates, has been widely used to filter out compounds with undesirable properties, especially those with poor ADMET-related profiles [16]. Natural products with inherently good biological properties are understandably receiving increasing attention [16]. Average DL values for Drugbank compounds ≥ 0.18 have been reported as a criterion for screening bioactive compounds in systems pharmacology-based analyses of TCM [12,17,18]. As shown in Table 1, the DL value of SAA was calculated to be 0.7 by TCMSP, which is above average, and therefore indicates that SAA may be a promising drug.

Target identification is the first step in drug discovery, and more and more drugs or active compounds are being shown to target multiple proteins [1,9,19,20]. Various in silico target fishing methods have been designed and are now widely used for this purpose [21]. As shown in Table 2, 13 putative targets of SAA were screened using computational tools.

Some of the putative SAA targets have been identified previously. For example, aldose reductase (AR) is a major mediator of inflammatory signals induced by oxidative stress, and plays a pivotal role in cellular metabolism, inflammation and cancer [22,23]. In vitro experiments showed that SAA inhibits AR activity and prevents galactose-induced cataract [24]. The GTPase HRas (Table 2) is a Ras isoform that, like other Ras proteins, functions as a GDP-GTP-regulated binary on-off switch that controls cytoplasmic signalling networks affecting a diverse range of cellular processes [25]. S-3-1, a synthetic intermediate of SAA, can suppress the overexpression of the c-myc oncogene, inhibit the function of the Ras oncoprotein, increase the expression of the P53 tumour suppressor, and interrupt P46-associated and mitogen-activated pathways distinct from farnesylation of Ras [26]. Glutathione S-transferases (GSTs) are a family of detoxification enzymes that catalyse the conjugation of glutathione to a wide variety of xenobiotics [27]. Danshensu, another active compound from Danshen, reduces MDA and restores GSH levels and total SH content in the cultured rat lens [28]. A combination of extracts from Danshen act pharmacologically via GST [29,30]. As for other putative targets, salvianolate, a highly concentrated form of salvianolic acid B, can inhibit neuronal apoptosis by increasing heat shock protein 22 in a reperfusion-ischemia model [31]. Therefore, the identification of 13 potential protein targets is consistent with multiple targets for SAA. The GeneMANIA results (Figure 3) provided information on co-expression and shared protein domains, and suggested that the targets and their interacting proteins may have identical or similar functions, in agreement with the results of the first step.

There have similar reports about predictive targets of SAA by computational tools. For example, Li developed an approach by combining network efficiency analysis with scoring function from molecular docking to estimate the anticoagulant activities of compounds which includes SAA [32]. A recently published paper also reported that SAA exhibits 8 hepatoprotective targets (BCL2, AKT1, CCND1, SPZ1, COL1A1, CDKN1A, HERC5 and MMP2) connections by in silico-based network pharmacology approach [33]. Notably, AKT1 and MMP3 are also the PharmMapper’s results of SAA in our study (Supporting Table S2).

GO and pathway analysis was also conducted using MAS 3.0, and a network was constructed using Cytoscape. As shown in Figure 4, the network also indicated that SAA has multiple targets and further implies that it possesses multiple pharmacological activities. Interestingly, HRAS was identified as a potential hub protein in the network (Figure 4). SAA reverses paclitaxel resistance in human breast cancer bytargeting the expression of transgelin2 and attenuating the PI3K/Akt pathway [34], inactivates transgelin2 [35], protects human neuroblastoma SH-SY5Y cells against MPP+-induced cytotoxicity [36] and inhibits the growth of A549 lung cancer cells [37]. These findings indicate that the anticancer activity of SAA likely involves Ras proteins.

## 4. Materials and Methods

### 4.1. Evaluation of Drug-Likeness Using the TCMSP Server

The TCMSP server (http://ibts.hkbu.edu.hk/LSP/tcmsp.php) is a systems-level pharmacology database for TCM that can also calculate ADME-related properties for interesting, naturally occurring compounds [12]. It provides an in silico ADME-systems evaluation model created by Wang’s research team, which integrated drug-likeness (DL), oral bioavailability (OB), and Caco-2 permeability and so on [12,17,18].

Drug-likeness (DL) is a qualitative concept used in drug design for an estimate on how “drug-like” a prospective compound is, which helps to optimize pharmacokinetic and pharmaceutical properties, such as solubility and chemical stability [17]. A database-dependent model is constructed based on the molecular descriptors and Tanimoto coefficient (as displayed below) [17,18]:

(1) $$T\;(A,\;B)\;=\;\frac{A\;\times \;B}{{\left\|A\right\|}^{2}+{\left\|B\right\|}^{2}-A\;\times \;B}$$

where A is the molecular descriptor of herbal ingredients, and B shows the average molecular properties of all molecules in Drug-Bank database [17,18].

Oral bioavailability (OB) is one of the most vital pharmacokinetic properties of orally administered drugs because it plays an important role for the efficiency of the drug delivery to the systemic circulation. Its value was calculated by an in-house model OBioavail1.1 in the TCMSP database [12,17].

For orally administered drugs, one of the greatest problems is movement across the intestinal epithelial barrier, which determines the rate and extent of human absorption and ultimately affects its bioavailability [12,17]. The Caco-2 permeability prediction model preCaco2 was applied in the TCMSP database [12,17].

In this study, the chemical name “salvianolic acid A” was entered as the search term and its druggability was analysed at the molecular level.

### 4.2. Computational Target Fishing by PharmMapper and DRAR-CPI

PharmMapper, a reverse docking server, can identify potential protein targets for small molecule compounds via a pharmacophore mapping approach [38]. For a given compound, it can provide the top 300 targets, sorted by fit score in descending order [38]. Meanwhile, the DRAR-CPI server can identify targetable proteins for small molecules via chemical-protein interactome analysis [39]. Both are powerful tools for computational target fishing.

An sdf file for SAA (PubChem CID: 5281793) was downloaded from the PubChem database and separately uploaded to the PharmMapper and DRAR-CPI servers. All parameters were set to default values, and overlapping protein targets identified using both servers were chosen for further investigation.

### 4.3. Analysis by GeneMANIA

GeneMANIA is a flexible, user-friendly web interface for generating hypotheses about gene function, analysing gene lists and prioritising genes for functional assays [40]. After selecting *Homo sapiens* from the nine optional organisms, the gene of interest in the previous step was entered into the search bar and the results were collated.

### 4.4. GO and Pathway Analysis, and Network Construction

The Molecule Annotation System 3.0 (MAS 3.0), a web-based software toolkit, can be used to help understand relationships within gene expression data and provide systematic and visual information on the gene of interest. Potential targets were uploaded to the MAS 3.0 server (http://bioinfo.capitalbio.com/mas3/) following the online instructions, and GO and KEGG pathway information for SAA was generated and collected. For a deeper understanding of the complex relationships among compounds, targets and diseases, networks were constructed and analysed using Cytoscape 3.0.

## 5. Conclusions

In the present study, the DL of SAA was evaluated by TCMSP, and potential targets identified by both PharmMapper and DRAR-CPI were chosen for further investigation. The results showed that SAA may be a good drug candidate, and 13 potential interacting partners were identified that are associated with various pharmacological activities. In addition, GO and pathway analysis was performed and a drug-target association network was constructed. These results indicated that SAA has multiple functions, including anticancer and metabolic activity. Although further studies are necessary to determine the precise interactions, this study provides a systematic and visual overview of possible SAA molecular mechanisms and signaling pathways.

## Figures and Tables

**Table 1:** Pharmacological and molecular properties of SAA.

Name	MW	AlogP	Hdon	Hacc	OB (%)	Caco-2	BBB	DL	FSAF	TPSA	RBN
SAA	494.48	4.14	7	10	2.96	−0.56	−1.62	0.7	0.4	184.98	9

**Table 2:** Putative targets of SAA identified by PharmMapper and DRAR-CPI.

Rank	PDB ID	Name	Target Gene
1	1T40	Aldose reductase	AKR1B1
2	2C3Q	Glutathione S-transferase theta-1	GSTT1
3	2BX8	Serum albumin	ALB
4	1LJR	Glutathione S-transferase theta-2	GSTT2B
5	1XMI	Cystic fibrosis transmembrane conductance regulator	CFTR
6	1EK5	UDP-glucose 4-epimerase	GALE
7	2E8A	Heat shock 70 kDa protein 1	HSPA1
8	1H0C	Serine-pyruvate aminotransferase	SPAT
9	11GS	Glutathione S-transferase P	GSTP1
10	5P21	GTPase HRas	HRAS
11	1A3B	Prothrombin	F2
12	1XWK	Glutathione S-transferase Mu 1	GSTM1
13	1R6T	Tryptophanyl-tRNA synthetase, cytoplasmic	WARS

**Table 3:** GO analysis of potential targets.

GO Term	Count	Percentage
GO:0007582 Physiological process	41	0.1348684211
GO:0009987 Cellular process	37	0.1217105263
GO:0005488 Binding	22	0.0723684211
GO:0065007 Biological regulation	20	0.0657894737
GO:0005623 Cell	19	0.0625
GO:0044464 Cell part	19	0.0625
GO:0050789 Regulation of biological process	17	0.0559210526
GO:0032501 Multicellular organismal process	16	0.0526315789
GO:0050896 Response to stimulus	16	0.0526315789
GO Other items	15	0.0493421053
GO:0003824 Catalytic activity	15	0.0493421053
GO:0008152 Metabolism	14	0.0460526316
GO:0032502 Developmental process	11	0.0361842105
GO:0051179 Localisation	10	0.0361842105
GO:0043226 Organelle	9	0.0296052632
GO:0051234 Establishment of localisation	8	0.0263157895
GO:0048519 Negative regulation of biological process	6	0.0197368421
GO:0048518 Positive regulation of biological process	5	0.0164473684
GO:0044422 Organelle part	4	0.0131578947

## Supplementary Materials

The following are available online. Table S1: KEGG pathways of putative targets identified by MAS 3.0, Table S2: The top 300 potential targets of SAA predicted by PharmMapper.
